# Supplementary material for: A multiple super-enhancer region establishes inter-TAD interactions and controls Hoxa function in cranial neural crest
Source: Nat Commun. 2023 Jun 5;14:3242. doi: 10.1038/s41467-023-38953-0 (PMC10241789; doi:10.1038/s41467-023-38953-0)
Supplement: Supplementary file 1 — Supplementary Information [file 41467_2023_38953_MOESM1_ESM.pdf]

A multiple super-enhancer region establishes inter-TAD interactions and controls *Hoxa* function in cranial neural crest

Kessler et al.

Supplementary Information

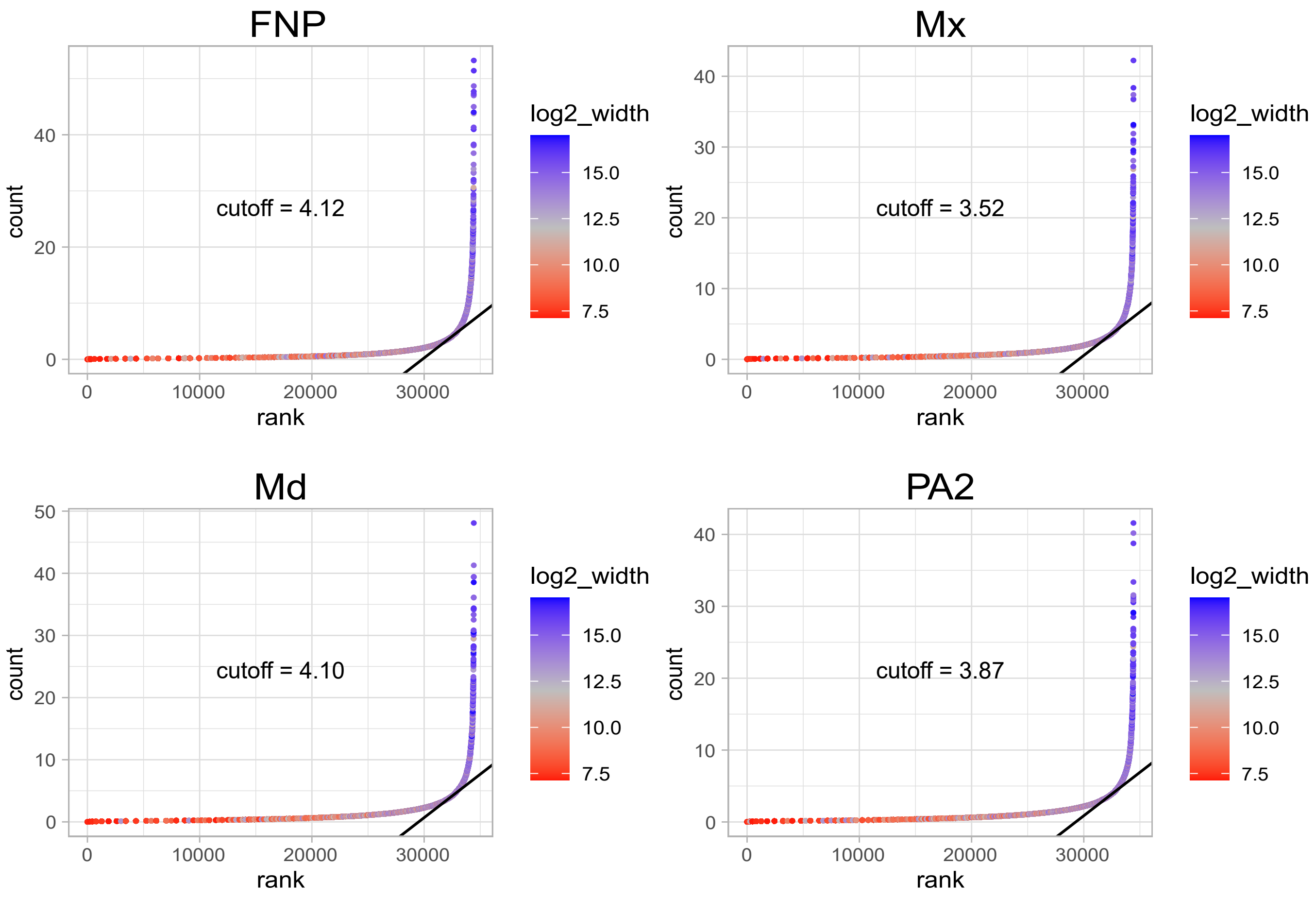
FNP
count
rank
cutoff = 4.12
log2_width
15.0
12.5
10.0
7.5
0
20
40
0
10000
20000
30000
Mx
cutoff = 3.52
0
10
20
30
40
Md
cutoff = 4.10
50
PA2
cutoff = 3.87

Supplementary Fig. 1 Identification of craniofacial super-enhancers.

Hockey stick plots displaying the H3K27 acetylation levels within merged peaks as counts per million on the y-axis and the according rank on the x-axis, separately for each cranial neural crest cell (CNCC) subpopulation. The cutoff value for the SE category was determined as described by the ROSE algorithm using the tangent (shown in black) and is indicated in the figure. The color reflects the log2 transformed width of the merged regions.

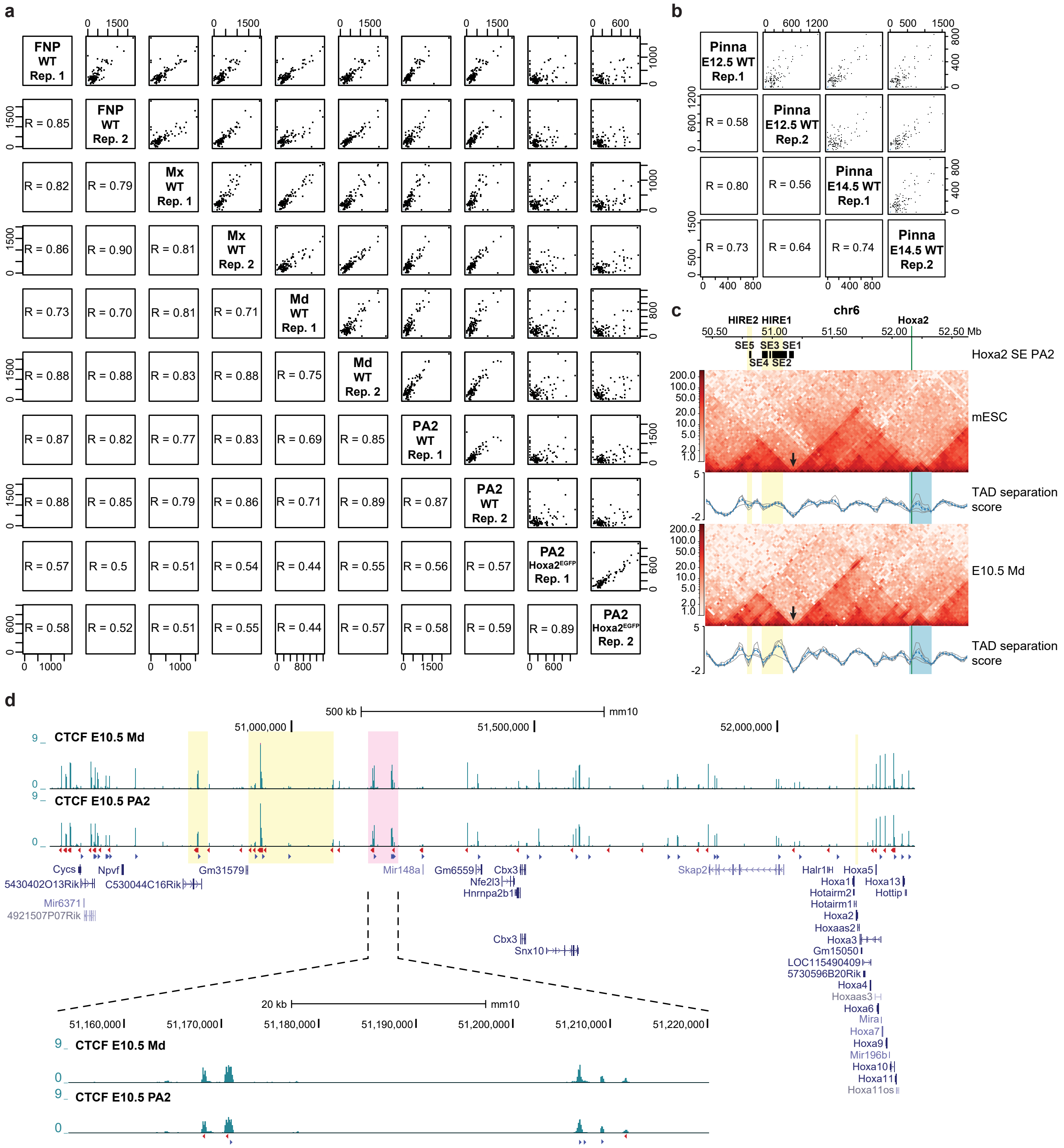
a
FNP WT Rep. 1
FNP WT Rep. 2
Mx WT Rep. 1
Mx WT Rep. 2
Md WT Rep. 1
Md WT Rep. 2
PA2 WT Rep. 1
PA2 WT Rep. 2
PA2 Hoxa2EGFP Rep. 1
PA2 Hoxa2EGFP Rep. 2
R = 0.85
R = 0.82
R = 0.79
R = 0.86
R = 0.90
R = 0.81
R = 0.73
R = 0.70
R = 0.81
R = 0.71
R = 0.88
R = 0.88
R = 0.83
R = 0.88
R = 0.75
R = 0.87
R = 0.82
R = 0.77
R = 0.83
R = 0.69
R = 0.85
R = 0.88
R = 0.85
R = 0.79
R = 0.86
R = 0.71
R = 0.89
R = 0.87
R = 0.57
R = 0.5
R = 0.51
R = 0.54
R = 0.44
R = 0.55
R = 0.56
R = 0.57
R = 0.58
R = 0.52
R = 0.51
R = 0.55
R = 0.44
R = 0.57
R = 0.58
R = 0.59
R = 0.89
b
Pinna E12.5 WT Rep.1
Pinna E12.5 WT Rep.2
Pinna E14.5 WT Rep.1
Pinna E14.5 WT Rep.2
R = 0.58
R = 0.80
R = 0.56
R = 0.73
R = 0.64
R = 0.74
c
chr6
HIRE2 HIRE1
Hoxa2
50.50 51.00 51.50 52.00 52.50 Mb
SE5 SE3 SE1
SE4 SE2
Hoxa2 SE PA2
mESC
TAD separation score
E10.5 Md
TAD separation score
d
500 kb
mm10
51,000,000
51,500,000
52,000,000
CTCF E10.5 Md
CTCF E10.5 PA2
Cycs
Npvf
Gm31579
Mir148a
Gm6559
Cbx3
Nfe2l3
Hnrnpa2b1
Skap2
Halr1
Hoxa5
Hoxa1
Hoxa13
Hotairm2
Hottip
Hotairm1
Hoxa2
Hoxaas2
Hoxa3
Gm15050
LOC115490409
5730596B20Rik
Hoxa4
Hoxaas3
Hoxa6
Mira
Hoxa7
Hoxa9
Mir196b
Hoxa10
Hoxa11
Hoxa11os
5430402O13Rik
C530044C16Rik
Mir6371
4921507P07Rik
Cbx3
Snx10
20 kb
mm10
51,160,000
51,170,000
51,180,000
51,190,000
51,200,000
51,210,000
51,220,000
CTCF E10.5 Md
CTCF E10.5 PA2

Supplementary Fig. 2 Promoter-capture Hi-C replicates, Hi-C in mESC and Md, and CTCF binding in PA2 and Md at E10.5.

a. Pairwise correlation scatter plots with Pearson's correlation coefficients between promoter capture Hi-C (PCHi-C) replicates from wild type (WT) frontonasal (FNP), maxillary (Mx), mandibular (Md), and second pharyngeal arch (PA2) derived cranial neural crest cells (CNCCs), as well as *Hoxa2*$^{EGFP/EGFP}$ PA2 derived CNCCs at E10.5. **b.** Pairwise correlation scatter plots with Pearson's correlation coefficients between PCHi-C replicates of pinna derived CNCCs at E12.5 and E14.5. **c.** Hi-C interaction heatmaps at 25 kb resolution in a 2.2 Mb region of chromosome 6 including the *Hoxa* cluster (50,442,417-52,636,150 bp) in mouse embryonic stem cells (mESC) and in Md derived CNCCs at E10.5. TAD separation scores are called with HiCExplorer's hicFindTADs and are shown as blue lines. The additional gray lines show the TAD score for different window sizes. The blue highlight marks the polycomb domain encompassing the *Hoxa* cluster and *Evx1* (chr6:52,145,433-52,327,518). Arrows indicate the location of the TAD boundary between *Hoxa2* and HIRE1/HIRE2. **d.** CTCF ChIP-seq tracks in Md and PA2 derived CNCCs at E10.5. CTCF motif orientation at CTCF peaks is indicated with red (minus strand) and blue (plus strand) arrowheads. The pink box highlights the CTCF binding sites at the TAD boundary between *Hoxa2* and HIRE1 / HIRE2. A zoom in on the TAD boundary is shown at the bottom. In panels **c** and d, yellow boxes highlight HIRE1/HIRE2 and a green (**c**) or yellow (**d**) line highlights the *Hoxa2* locus.

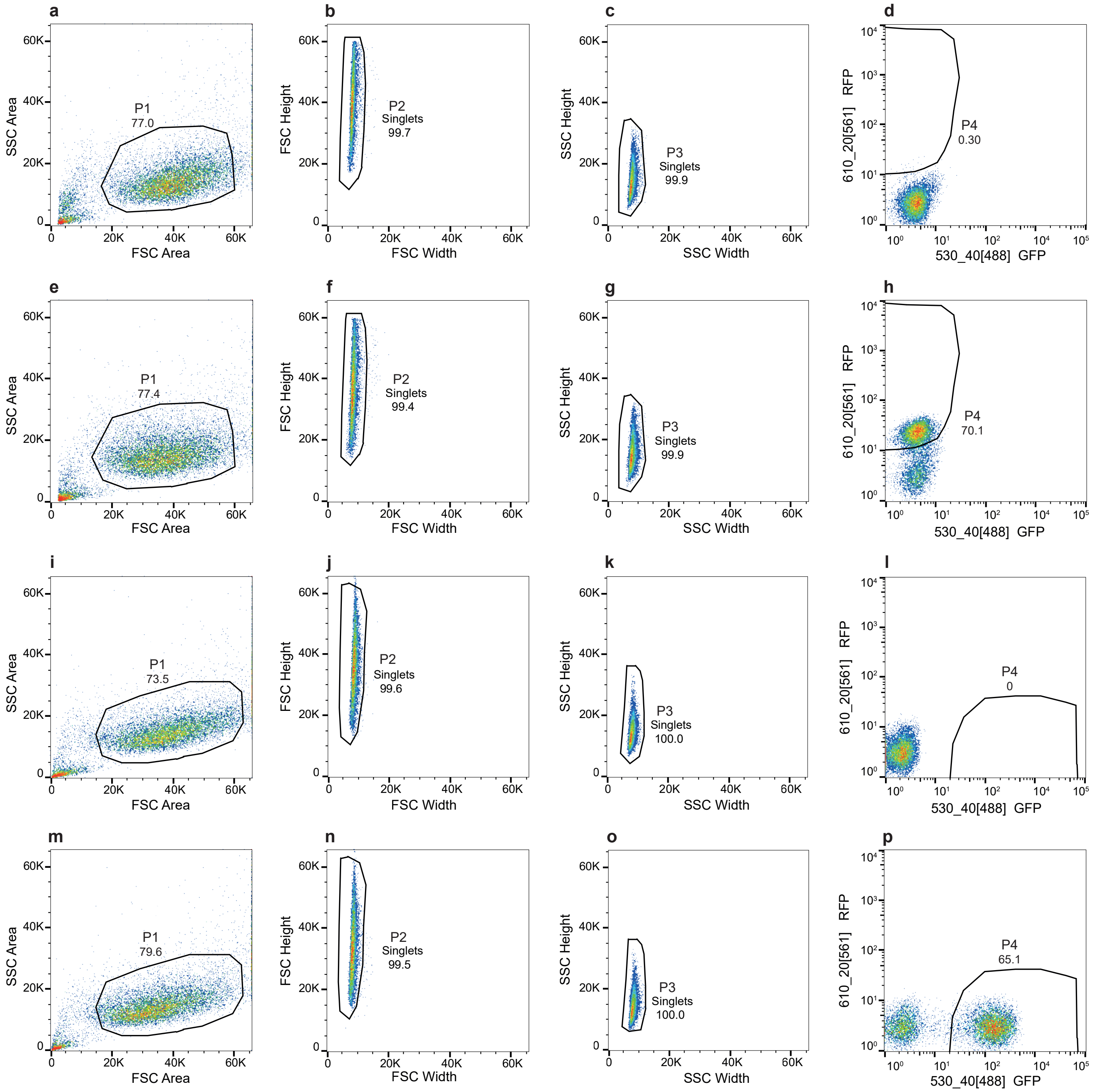
a
SSC Area
FSC Area
P1
77.0
b
FSC Height
FSC Width
P2
Singlets
99.7
c
SSC Height
SSC Width
P3
Singlets
99.9
d
610_20[561] RFP
530_40[488] GFP
P4
0.30
e
SSC Area
FSC Area
P1
77.4
f
FSC Height
FSC Width
P2
Singlets
99.4
g
SSC Height
SSC Width
P3
Singlets
99.9
h
610_20[561] RFP
530_40[488] GFP
P4
70.1
i
SSC Area
FSC Area
P1
73.5
j
FSC Height
FSC Width
P2
Singlets
99.6
k
SSC Height
SSC Width
P3
Singlets
100.0
l
610_20[561] RFP
530_40[488] GFP
P4
0
m
SSC Area
FSC Area
P1
79.6
n
FSC Height
FSC Width
P2
Singlets
99.5
o
SSC Height
SSC Width
P3
Singlets
100.0
p
610_20[561] RFP
530_40[488] GFP
P4
65.1

Supplementary Fig. 3 Fluorescence activated cell sorting (FACS) gating strategy.

a-p. FACS of Red Fluorescent Protein (RFP)-positive cranial neural crest cells (CNCCs) from *Wnt1::Cre*;*ROSA*^{RFP}embryos (**a-h**) and FACS of Green Fluorescent Protein (GFP)-positive cells from the second arch of E10.5*Hoxa2*$^{EGFP/EGFP}$ embryos (**i-p**). Living cells are first discriminated from debris by scatter areas P1 (FSC Area versus SSC Area) (**a**, **e**, **i**, **m**). Single cells are discriminated from doublets by firstly assessing forward scatter (FSC Width versus FSC Height; P2) (**b**, **f**, **j**, **n**) and next by assessing side scatter (SSC Width versus SSC Height; P3) (**c**, **g**, **k**, **o**). Subsequently, cells were sorted based on fluorescent markers of interest, GFP (**h**) or RFP (**p**). The baseline control fluorescent signal is from a wild-type (**d**, **l**) littermate (negative control).

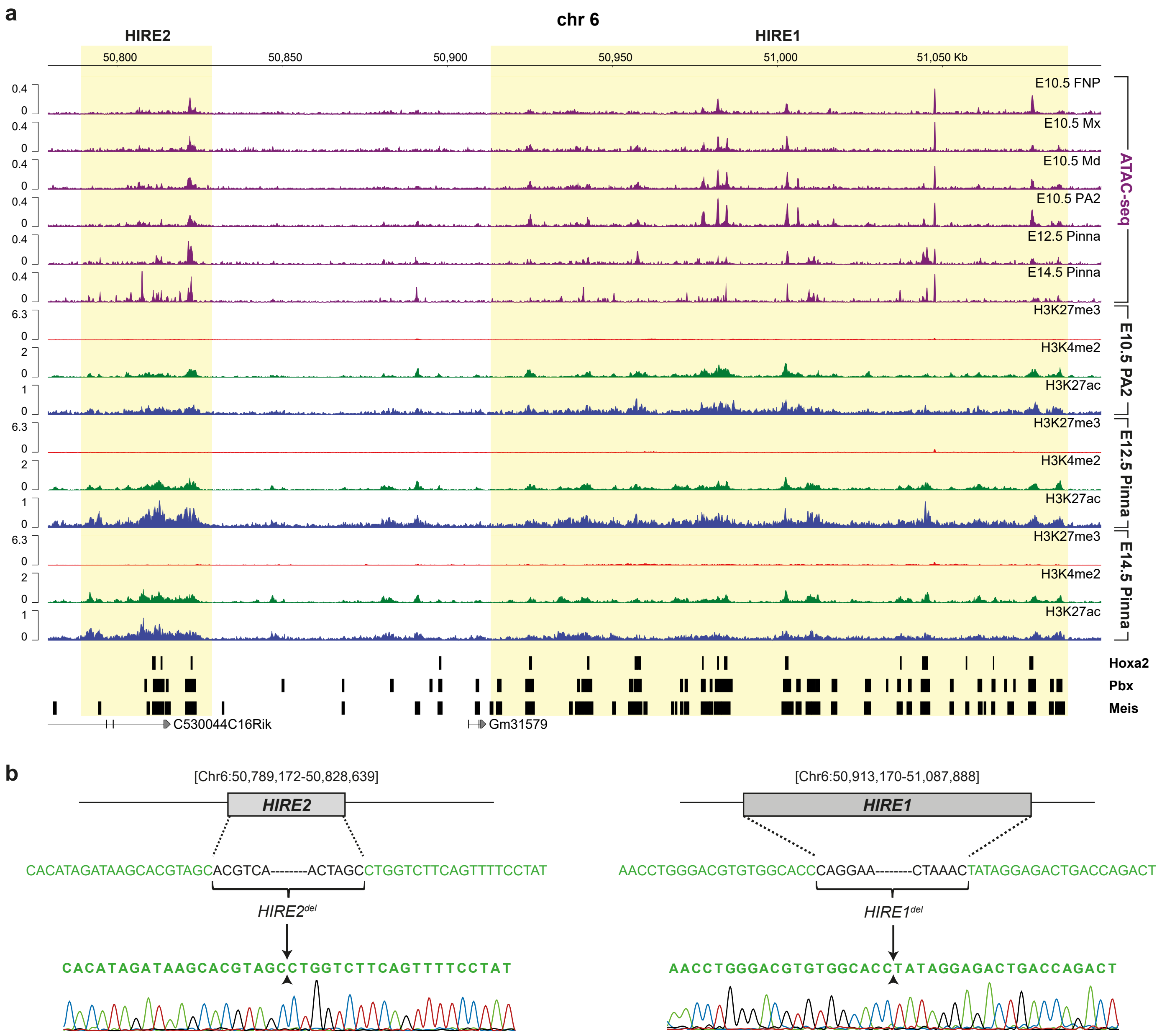

a
chr 6
HIRE2
HIRE1
50,800
50,850
50,900
50,950
51,000
51,050 Kb
E10.5 FNP
E10.5 Mx
E10.5 Md
E10.5 PA2
E12.5 Pinna
E14.5 Pinna
ATAC-seq
H3K27me3
H3K4me2
H3K27ac
E10.5 PA2
E12.5 Pinna
E14.5 Pinna
Hoxa2
Pbx
Meis
C530044C16Rik
Gm31579
b
[Chr6:50,789,172-50,828,639]
HIRE2
CACATAGATAAGCACGTAGCACGTCA--------ACTAGCCTGGTCTTCAGTTTTCCTAT
HIRE2del
CACATAGATAAGCACGTAGCCTGGTCTTCAGTTTTCCTAT
[Chr6:50,913,170-51,087,888]
HIRE1
AACCTGGGACGTGTGGCACCCAGGAA--------CTAAACTATAGGAGACTGACCAGACT
HIRE1del
AACCTGGGACGTGTGGCACCTATAGGAGACTGACCAGACT

Supplementary Fig. 4 Chromatin landscape of HIRE1 and HIRE2.

a. Genome browser view on HIRE1 and HIRE2 (chr6:50,779,104 – 51,097,965) with chromatin accessibility (ATAC-seq, purple) from the frontonasal process (FNP), the maxillary (Mx), the mandibular (Md) and the second pharyngeal arch (PA2) cranial neural crest cells (CNCCs) at E10.5, and from the pinna CNCCs at E12.5 and E14.5. ChIP-seq profiles for H3K27me3 (red), H3K4me2 (green), and H3K27ac (blue) from PA2 CNCCs at E10.5, and from the pinna CNCCs at E12.5 and E14.5 are also shown. At the bottom are binding sites identified by ChIP-seq, for Hoxa2[1], Pbx, and Meis[2] in PA2 at E11.5. Highlighted in yellow are HIRE1 and HIRE2. **b.** Illustration representing HIRE1 [Chr6:50,913,170-51,087,888] and HIRE2 [Chr6:50,789,172-50,828,639] deletions. HIRE1 and HIRE2 regions were deleted in mutant mice using the CRISPR-Cas9 system, with regions preserved after deletion represented in green, and regions deleted represented in black. $HIRE1^{del}$ and $HIRE2^{del}$ mutations were validated using sanger sequencing.

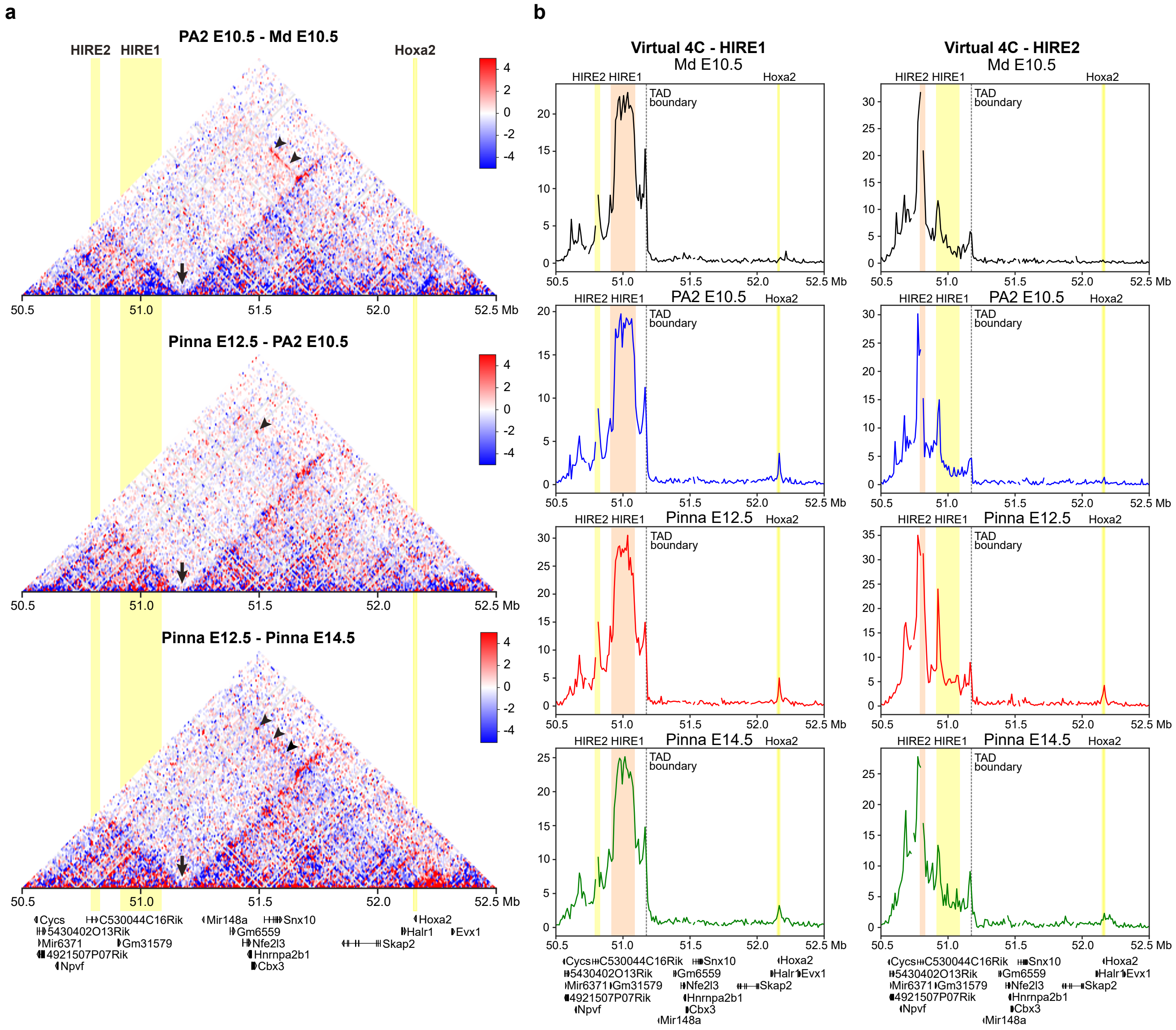

a
PA2 E10.5 - Md E10.5
HIRE2
HIRE1
Hoxa2
Pinna E12.5 - PA2 E10.5
Pinna E12.5 - Pinna E14.5
50.5
51.0
51.5
52.0
52.5 Mb
Cycs
C530044C16Rik
5430402O13Rik
Mir6371
Gm31579
4921507P07Rik
Npvf
Mir148a
Snx10
Gm6559
Nfe2l3
Hnrnpa2b1
Cbx3
Hoxa2
Halr1
Evx1
Skap2
b
Virtual 4C - HIRE1
Virtual 4C - HIRE2
Md E10.5
PA2 E10.5
Pinna E12.5
Pinna E14.5
TAD boundary

Supplementary Fig. 5 Interactions at HIRE1 and HIRE2

a. Differential HiC maps at 10 kb resolution are shown for a ~2Mb region of chromosome 6 including the *Hoxa* cluster, comparing interactions between E10.5 second pharyngeal arch (PA2) and E10.5 mandibular (Md) cranial neural crest cells (CNCCs) (top), between E12.5 pinna CNCCs and E10.5 PA2 CNCCs (middle), and between E12.5 pinna CNCCs and E14.5 pinna CNCCs (bottom). Shown below are the positions of genes. For better readability, only the genomic position of the *Hoxa2* gene is shown within the *Hoxa* cluster. The yellow rectangles show the HIRE1/HIRE2 regions and *Hoxa2* gene. The arrowheads on the differential map highlight the "stripe" or "spots" of increased interactions between *Hoxa2* and HIRE1/HIRE2 (red) in E10.5 PA2 as compared to Md (top) in E12.5 pinna as compared to E10.5 PA2 (middle) and in E12.5 pinna as compared to E14.5 pinna (bottom). A black arrow between two TADs indicates the presence of the TAD border. **b.** Virtual 4C plots from HIRE1 (left side) and HIRE2 (right side) for E10.5 Md, E10.5 PA2, E12.5 pinna and E14.5 pinna CNCCs, derived from Hi-C data. The orange rectangles highlight the viewpoints whereas the yellow rectangles highlight the *Hoxa2* gene, HIRE2 (left side) and HIRE1 (right side).

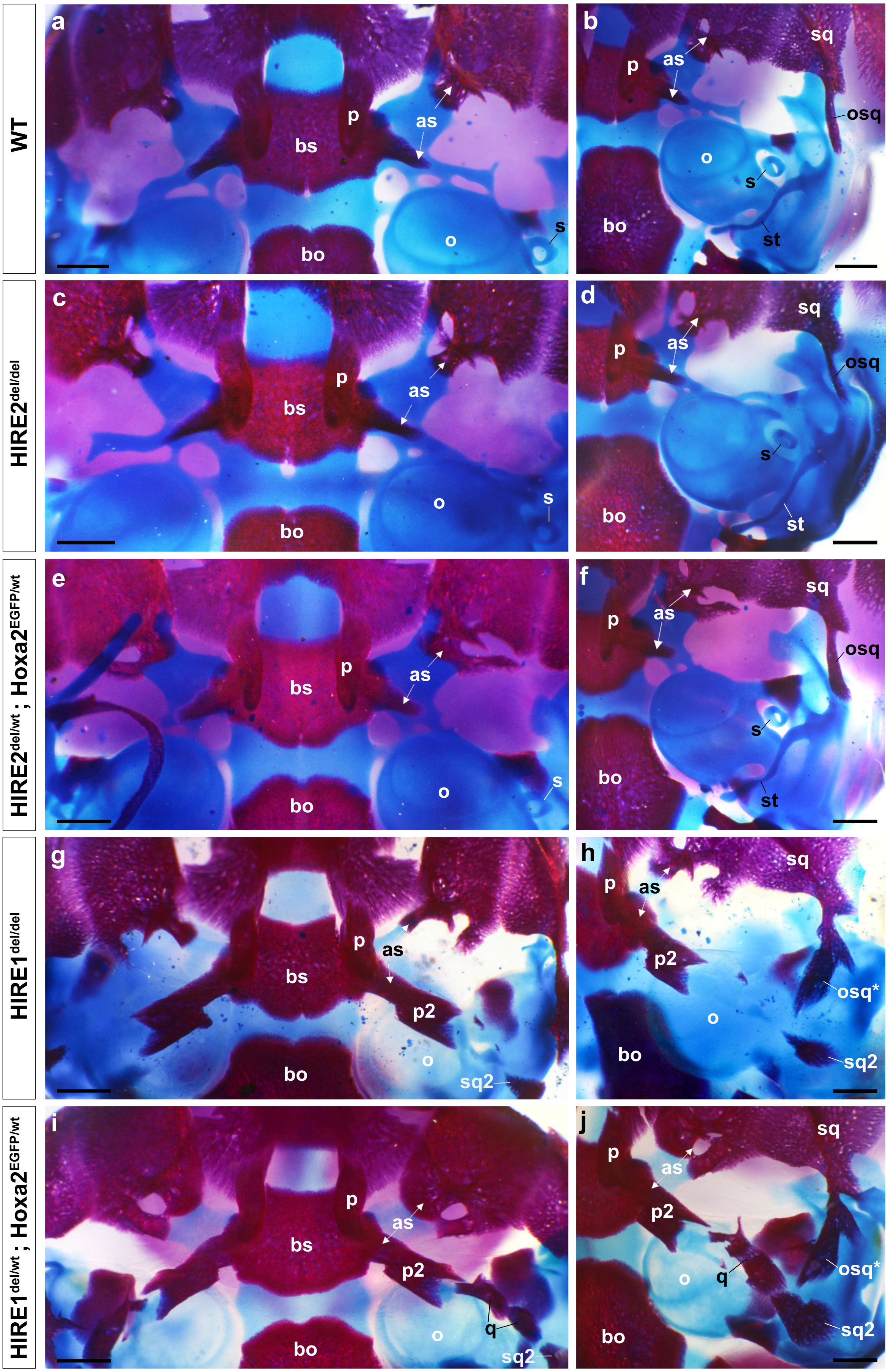

WT
HIRE2del/del
HIRE2del/wt ; Hoxa2EGFP/wt
HIRE1del/del
HIRE1del/wt ; Hoxa2EGFP/wt
a
b
c
d
e
f
g
h
i
j
p
as
bs
bo
o
s
sq
osq
st
p2
sq2
osq*
q

Supplementary Fig. 6 Cranial skeletal changes in HIRE1 and HIRE2 mutant fetuses.

Ventral (**a, c, e, g, i**) and ventrolateral (**b, d, f, h, j**) views of the cranial base of E18.5 wild-type (WT) **(a, b**), *HIRE2*$^{del/del}$ homozygous mutant (**c, d**), *HIRE2*$^{del/wt}$*;Hoxa2*$^{EGFP/wt}$ trans-heterozygote mutant (**e, f**), *HIRE1*$^{del/del}$ homozygous mutant (**g, h**) and *HIRE1*$^{del/wt}$*;Hoxa2*$^{EGFP/wt}$ trans-heterozygous mutant (**i, j**) fetuses. **a** to **j** are representative images of n=4/4 WT (**a**, **b**), n=6/6 *HIRE2*$^{del/del}$ (**c, d**), n=4/4 *HIRE2*$^{del/wt}$*;Hoxa2*$^{EGFP/wt}$ (**e**, **f**), n=4/4 *HIRE1*$^{del/del}$ (**g**, **h**) and n=4/4 *HIRE1*$^{del/wt}$*;Hoxa2*$^{EGFP/wt}$ (**i**, **j**) fetuses. as represents the alisphenoid bone; bo represents the basioccipital bone; bs represents the basisphenoid bone; o represents the cartilaginous otic capsule; p represents the pterygoid bone; p2 represents the ectopic pterygoid bone; osq and osq* represent respectively the normal and modified retrotympanic (otic) process of the squamosal bone; s represents the stapes; sq represents the squamosal bone; sq2 represents the ectopic squamosal bone; st represents the styloid process; q represents the atavistic quadrate bone, ectopically appearing in *Hoxa2*$^{-/-}$ mutants[3] and in the *HIRE1*$^{del/wt}$*;Hoxa2*$^{EGFP/wt}$ trans-heterozygous mutants. Scale bars represent 500 µm.

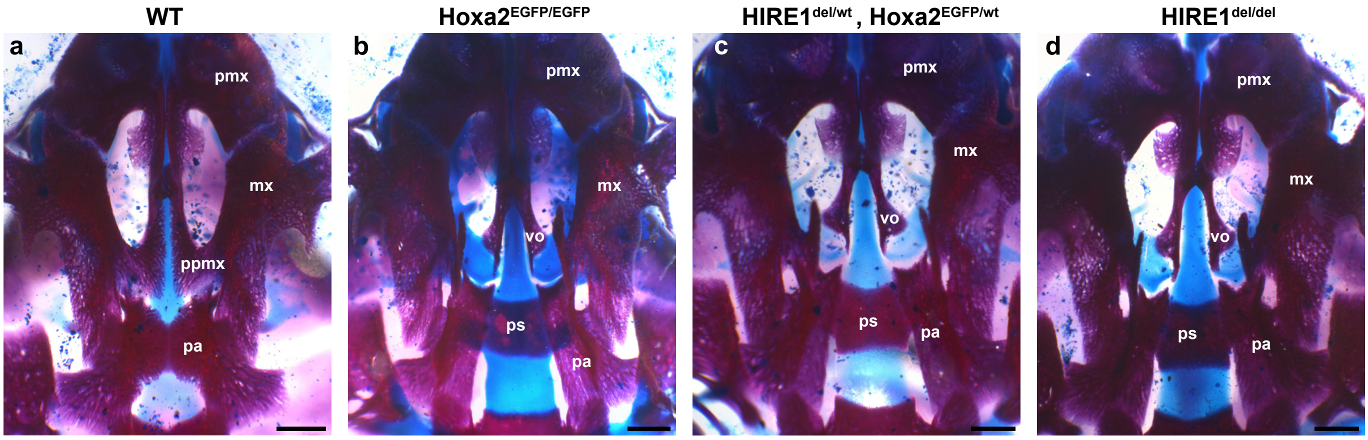
WT
Hoxa2EGFP/EGFP
HIRE1del/wt, Hoxa2EGFP/wt
HIRE1del/del
a
pmx
mx
ppmx
pa
b
pmx
mx
vo
ps
pa
c
pmx
mx
vo
ps
pa
d
pmx
mx
vo
ps
pa

Supplementary Fig. 7 Cleft secondary palate in HIRE1 mutant fetuses

Skeletal preparations of the palate area after the removal of the lower jaw from E18.5 wild-type (WT) (**a**), *Hoxa2*$^{EFGP/EGFP}$ (**b**), *HIRE1*$^{del/wt}$;*Hoxa2*$^{EGFP/wt}$ (**c**) and *HIRE1*$^{del/del}$ (**d**) fetuses. **a** to **d** are representative images of WT (n=4/4) (**a**), *Hoxa2*$^{EFGP/EGFP}$ (n=6/8) (**b**), *HIRE1*$^{del/wt}$;*Hoxa2*$^{EGFP/wt}$ (n=14/24) (**c**) and *HIRE1*$^{del/del}$ (n=8/10) (**d**) fetuses. The nose is toward the top. mx represents the maxilla, pa the palatal bone, pmx the premaxilla, ppmx the palatal process of maxilla, ps the presphenoid bone, and vo the vomer. Note that vo and ps in (b, c, d) are not visible in (a) because they are underneath Mx and Pa. Scale bars represent 500 µm.

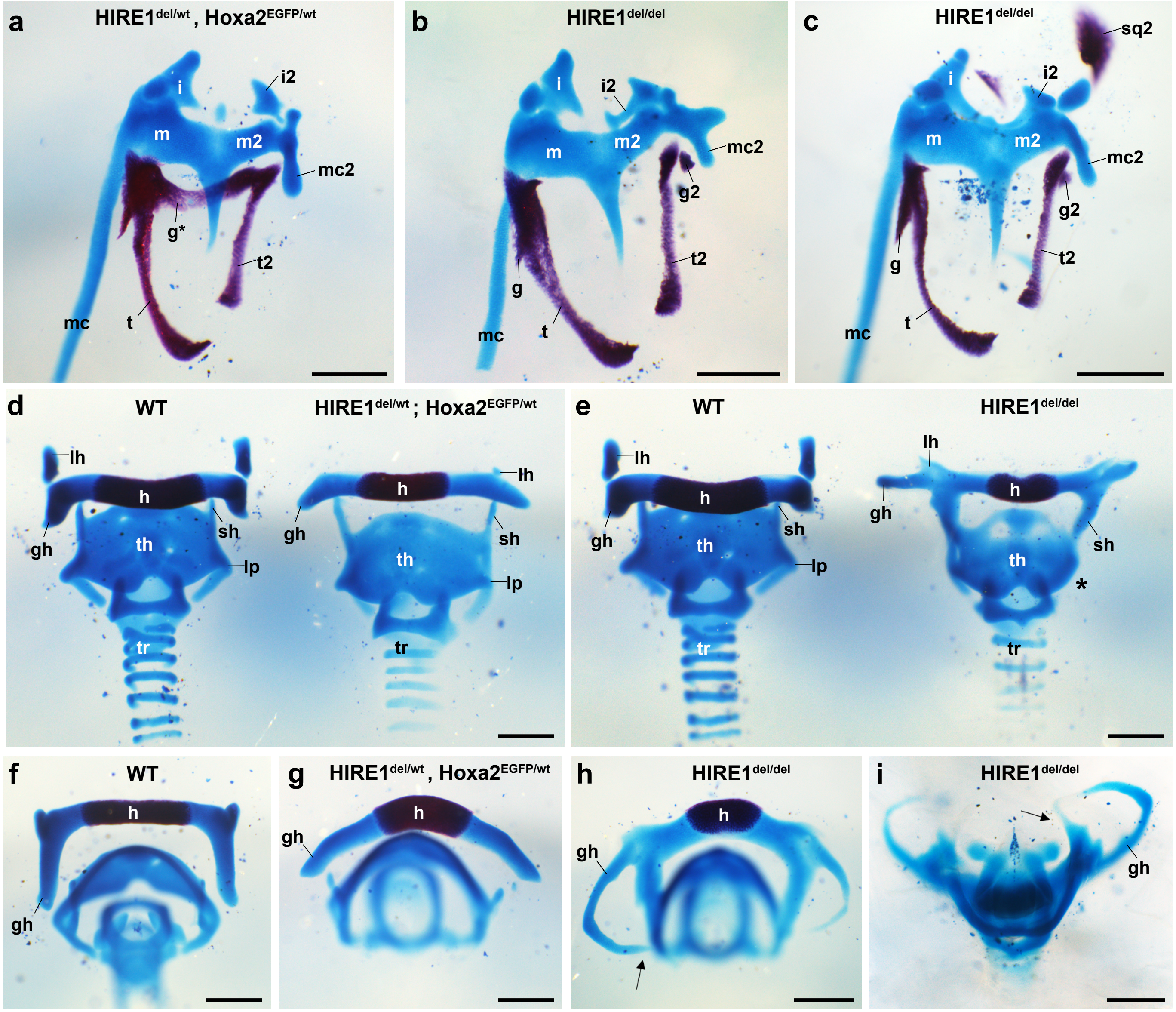

a
HIRE1del/wt, Hoxa2EGFP/wt
i
i2
m
m2
mc2
g*
t2
mc
t
b
HIRE1del/del
i
i2
m
m2
mc2
g2
t2
g
mc
t
c
HIRE1del/del
sq2
i
i2
m
m2
mc2
g2
g
t2
mc
t
d
WT
HIRE1del/wt; Hoxa2EGFP/wt
lh
h
sh
gh
th
lp
tr
e
WT
HIRE1del/del
lh
h
sh
gh
th
lp
tr
*
f
WT
h
gh
g
HIRE1del/wt, Hoxa2EGFP/wt
h
gh
h
HIRE1del/del
h
gh
i
HIRE1del/del
gh

Supplementary Fig. 8 Middle ear and hyoid bone skeletal phenotypes upon deletion of HIRE1

Middle ear (**a-c**) and hyoid (**d-i**) skeletal preparations from E18.5 wild-type (WT) (**d, f**), *HIRE1*$^{del/del}$ homozygous mutant (**b, c, e, h, i**) and *HIRE1*$^{del/wt}$*;Hoxa2*$^{EGFP/wt}$ trans-heterozygous mutant (**a, d, g**) fetuses. In **a** and **b**, the absence of fusion between the incus and its duplicated counterpart is representative of n=1/8 fetus sides for *HIRE1*$^{del/wt}$*;Hoxa2*$^{EGFP/wt}$ (**a**) and n=1/8 fetus sides for *HIRE1*$^{del/del}$ (**b**). g* represents the transformed gonial bone, as described in *Hoxa2-/-* mutant fetuses [3]. The transformed gonial bone presented in **a** is representative of n=2/8 *HIRE1*$^{del/wt}$*;Hoxa2*$^{EGFP/wt}$ fetus sides. **d**, **i** and **j** are representative of WT (n=4/4), *HIRE1*$^{del/wt}$*;Hoxa2*$^{EGFP/wt}$ (n=4/4) and *HIRE1*$^{del/del}$ (n=4/4) fetuses._In **e**, the asterisk displays the absence of the lateral process (lp) of the laryngeal cartilage in *HIRE1*$^{del/del}$ homozygous mutant fetuses. The black arrows in **h** and **i** display the fusion between the elongated greater horn (gh) of the hyoid bone and the posterior border of the thyroid cartilage (th) observed in *HIRE1*$^{del/del}$ homozygous mutant fetuses. g and g2 represent the WT and duplicated gonial bones, respectively; h represents the body of the hyoid bone; i and i2 represent the WT and duplicated incus respectively; lh represents the lesser horn of the hyoid bone; m and m2 represent the WT and duplicated mallei, respectively; mc and mc2 represent the WT and partially duplicated Meckel's cartilages respectively; sh represents the superior horn of the thyroid cartilage; sq2 represents the ectopic squamosal bone; t and t2 represent the WT and duplicated tympanic bones respectively; tr represents the trachea. Scale bars represent 500 µm.

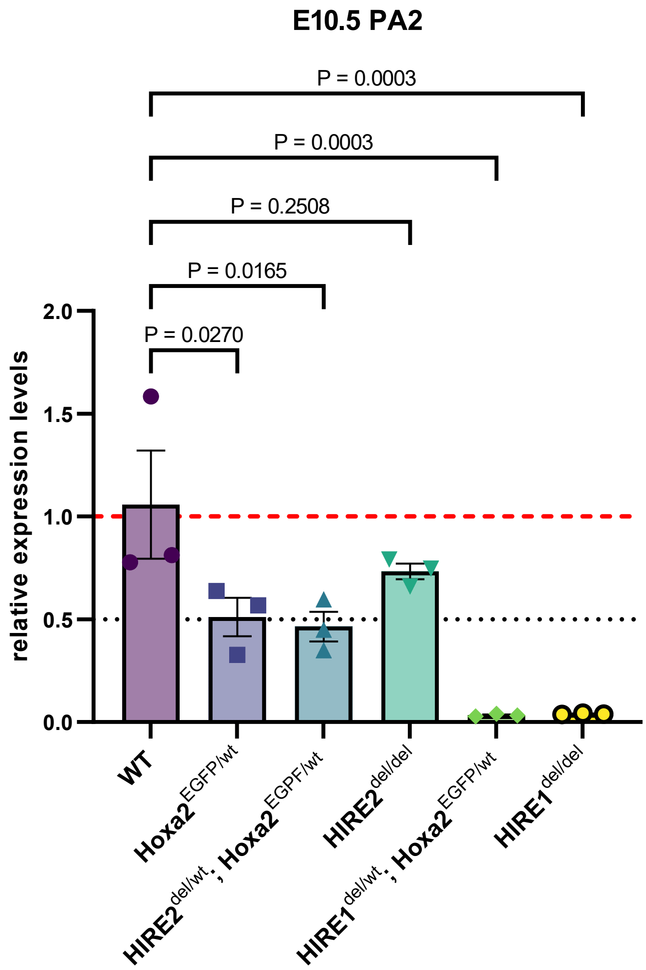
E10.5 PA2
P = 0.0003
P = 0.0003
P = 0.2508
P = 0.0165
P = 0.0270
relative expression levels
2.0
1.5
1.0
0.5
0.0
WT
Hoxa2EGFP/wt
HIRE2del/wt; Hoxa2EGPF/wt
HIRE2del/del
HIRE1del/wt; Hoxa2EGFP/wt
HIRE1del/del

Supplementary Fig. 9 *Hoxa2* down-regulation in E10.5 second pharyngeal arch upon HIRE1 or HIRE2 deletion.

Relative expression levels of *Hoxa2*, detected by qRT-PCR analysis, in E10.5 second pharyngeal arch (PA2) of WT and $Hoxa2^{EGFP/wt}$, $HIRE2^{del/wt}$;$Hoxa2^{EGFP/wt}$, $HIRE2^{del/del}$, $HIRE1^{del/wt}$;$Hoxa2^{EGFP/wt}$, and $HIRE1^{del/del}$ mutant embryos (n = 3 per genotype). Data are represented as means ± SEM. Significance is based on a one-way ANOVA followed by Dunnett's multiple comparisons test with WT as the control sample.

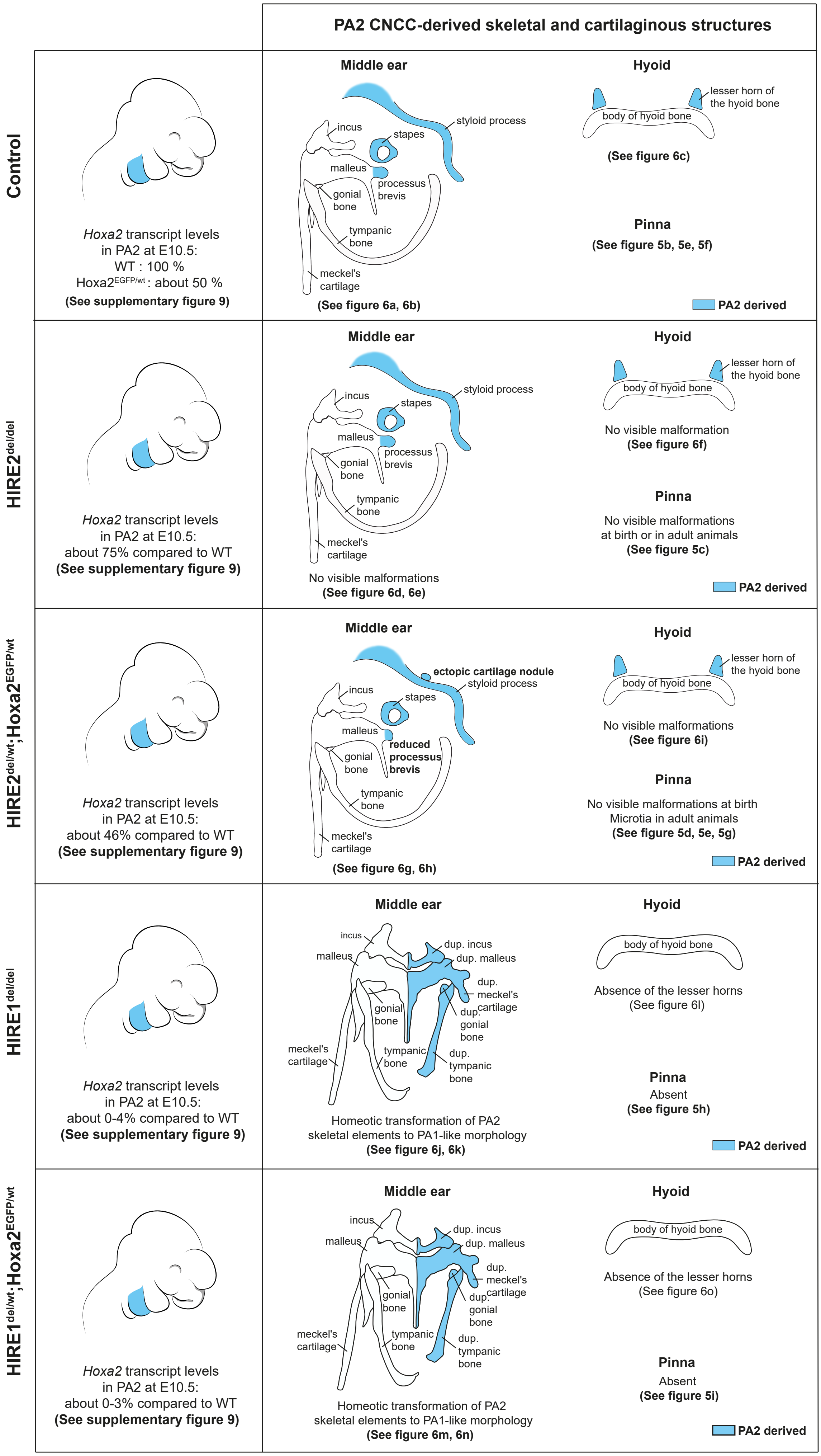
PA2 CNCC-derived skeletal and cartilaginous structures
Control
Hoxa2 transcript levels in PA2 at E10.5:
WT : 100 %
$Hoxa2^{EGFP/wt}$: about 50 %
(See supplementary figure 9)
Middle ear
incus
stapes
styloid process
malleus
processus brevis
gonial bone
tympanic bone
meckel's cartilage
(See figure 6a, 6b)
Hyoid
lesser horn of the hyoid bone
body of hyoid bone
(See figure 6c)
Pinna
(See figure 5b, 5e, 5f)
PA2 derived
$HIRE2^{del/del}$
Hoxa2 transcript levels in PA2 at E10.5:
about 75% compared to WT
(See supplementary figure 9)
Middle ear
incus
stapes
styloid process
malleus
processus brevis
gonial bone
tympanic bone
meckel's cartilage
No visible malformations
(See figure 6d, 6e)
Hyoid
lesser horn of the hyoid bone
body of hyoid bone
No visible malformation
(See figure 6f)
Pinna
No visible malformations at birth or in adult animals
(See figure 5c)
PA2 derived
$HIRE2^{del/wt};Hoxa2^{EGFP/wt}$
Hoxa2 transcript levels in PA2 at E10.5:
about 46% compared to WT
(See supplementary figure 9)
Middle ear
ectopic cartilage nodule
styloid process
incus
stapes
malleus
reduced processus brevis
gonial bone
tympanic bone
meckel's cartilage
(See figure 6g, 6h)
Hyoid
lesser horn of the hyoid bone
body of hyoid bone
No visible malformations
(See figure 6i)
Pinna
No visible malformations at birth
Microtia in adult animals
(See figure 5d, 5e, 5g)
PA2 derived
$HIRE1^{del/del}$
Hoxa2 transcript levels in PA2 at E10.5:
about 0-4% compared to WT
(See supplementary figure 9)
Middle ear
incus
malleus
dup. incus
dup. malleus
dup. meckel's cartilage
gonial bone
dup. gonial bone
meckel's cartilage
tympanic bone
dup. tympanic bone
Homeotic transformation of PA2 skeletal elements to PA1-like morphology
(See figure 6j, 6k)
Hyoid
body of hyoid bone
Absence of the lesser horns
(See figure 6l)
Pinna
Absent
(See figure 5h)
PA2 derived
$HIRE1^{del/wt};Hoxa2^{EGFP/wt}$
Hoxa2 transcript levels in PA2 at E10.5:
about 0-3% compared to WT
(See supplementary figure 9)
Middle ear
incus
malleus
dup. incus
dup. malleus
dup. meckel's cartilage
gonial bone
dup. gonial bone
meckel's cartilage
tympanic bone
dup. tympanic bone
Homeotic transformation of PA2 skeletal elements to PA1-like morphology
(See figure 6m, 6n)
Hyoid
body of hyoid bone
Absence of the lesser horns
(See figure 6o)
Pinna
Absent
(See figure 5i)
PA2 derived

Supplementary Fig. 10 Morphological changes of second arch-derived skeletal and cartilaginous structures correlate to reduction of *Hoxa2* transcript levels.

(Left) *Hoxa2* transcript levels in E10.5 second pharyngeal arch (PA2) of *Hoxa2*$^{EGFP/wt}$, *HIRE2*$^{del/del}$, *HIRE2*$^{del/wt}$;*Hoxa2*$^{EGFP/wt}$, *HIRE1*$^{del/del}$ and *HIRE1*$^{del/wt}$;*Hoxa2*$^{EGFP/wt}$ mutant embryos, as compared to wild-type (WT), and obtained by qRT-PCR analysis (see suppl. fig. 9). (Right) For each genotype, the corresponding phenotype at PA2 cranial neural crest cells (CNCC)-derived skeletal and cartilaginous structures (blue) is represented. Note that no visible malformations are observed in *Hoxa2*$^{EGFP/wt}$ fetuses.

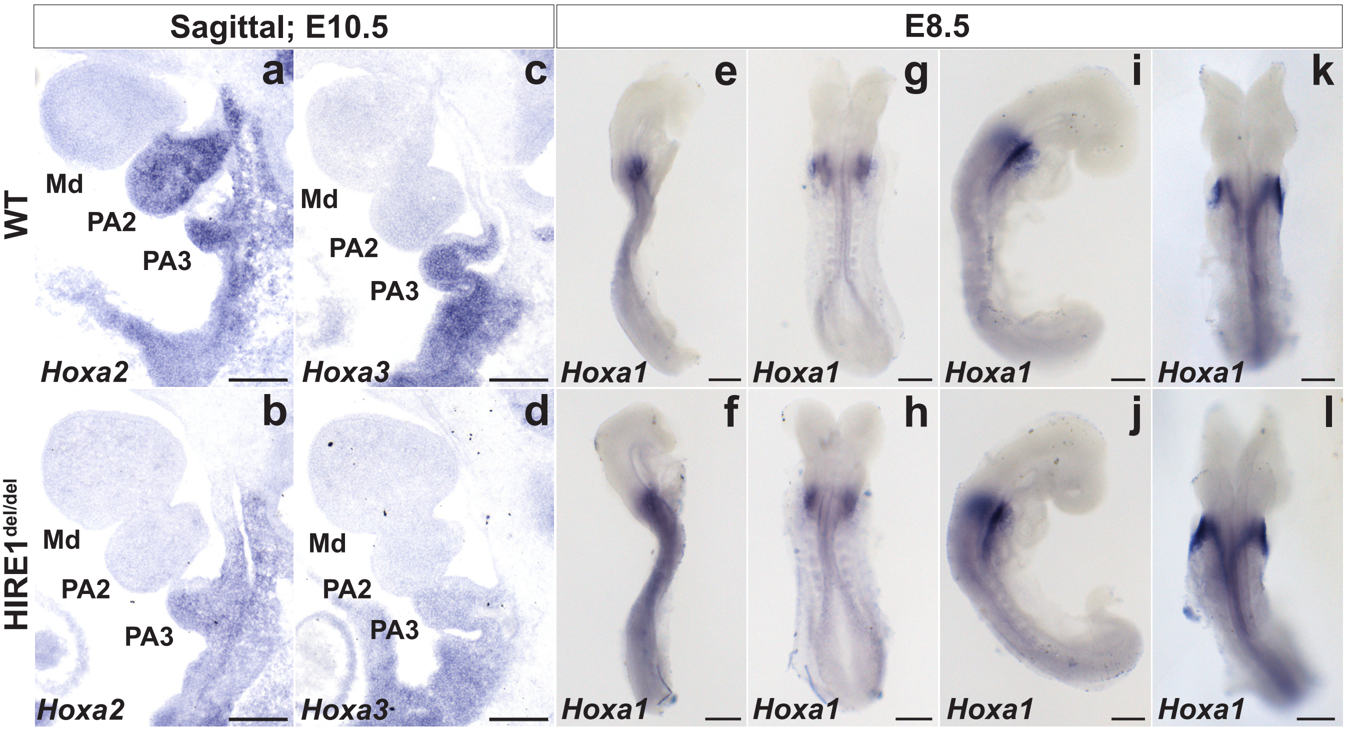
Sagittal; E10.5
E8.5
WT
HIRE1$^{del/del}$
a
b
c
d
e
f
g
h
i
j
k
l
Md
PA2
PA3
Hoxa2
Hoxa3
Hoxa1

Supplementary Fig. 11 Effect of HIRE1 deletion on anterior *Hoxa* gene expression.

In-situ hybridization on sagittal sections of wild-type (WT) (**a**, **c**) and $HIRE1^{del/del}$ (**b**, **d**) embryos at E10.5 using *Hoxa2* (**a**, **b**) and *Hoxa3* (**c**, **d**) antisense probes. **a-d** are representative images of serial sections from n=4 WT and n=4 $HIRE1^{del/del}$ embryos. *Hoxa2* expression is undetectable in the second pharyngeal arch (PA2) and strongly reduced in the third pharyngeal arch (PA3) of $HIRE1^{del/del}$ embryos (**b**) compared to WT (**a**). *Hoxa3* expression is strongly reduced in PA3 of $HIRE1^{del/del}$ embryos (**d**) compared to WT (**c**). Whole mount *in-situ* hybridization on WT (**e, g, i, k**; n=5 embryos) and $HIRE1^{del/del}$ (**f, h, j, l**; n=2 embryos) embryos at E8.5 using *Hoxa1* antisense probes. No differences in *Hoxa1* expression could be observed between WT and $HIRE1^{del/del}$ embryos. Md represents the mandibular process. Scale bars represent 200 µm.

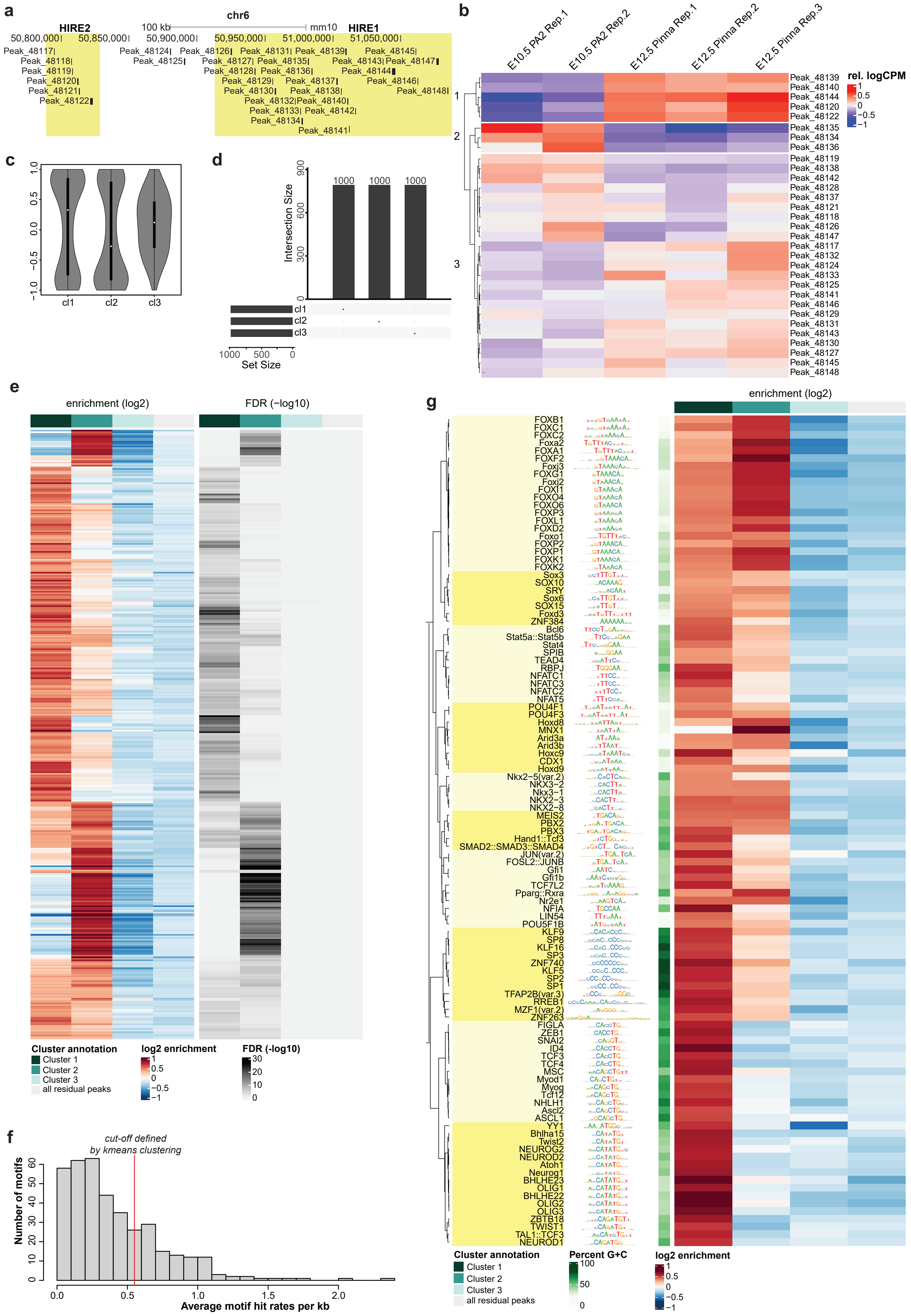
a
chr6
100 kb
mm10
HIRE2
HIRE1
50,800,000
50,850,000
50,900,000
50,950,000
51,000,000
51,050,000
b
E10.5 PA2 Rep.1
E10.5 PA2 Rep.2
E12.5 Pinna Rep.1
E12.5 Pinna Rep.2
E12.5 Pinna Rep.3
rel. logCPM
c
cl1
cl2
cl3
d
Intersection Size
Set Size
cl1
cl2
cl3
e
enrichment (log2)
FDR (−log10)
Cluster annotation
Cluster 1
Cluster 2
Cluster 3
all residual peaks
log2 enrichment
FDR (-log10)
f
cut-off defined by kmeans clustering
Number of motifs
Average motif hit rates per kb
g
enrichment (log2)
Cluster annotation
Cluster 1
Cluster 2
Cluster 3
all residual peaks
Percent G+C
log2 enrichment

Supplementary Fig.12 Transcription factor motif enrichment analysis.

a. Schematic representation of a genomic window, depicting the locations and names of 31 merged ATAC-seq peaks overlapping HIRE1 and HIRE2 from the second arch (PA2) derived CNCCs at E10.5 and from pinna derived CNCCs at E12.5. **b.** Clustering of ATAC-seq peaks overlapping HIRE1 and HIRE2 (chr6:50789172-51087888, mouse GRCm38/mm10) from PA2 derived CNCCs at E10.5 and from pinna derived CNCCs at E12.5 according to their relative accessibility levels at E10.5 and E12.5. Peaks were classified into three groups using hierarchical clustering. **c.** Violin plot depicting the distribution of Pearson's correlation coefficients of the ATAC-seq signal at all ATAC-seq peaks in E10.5 and E12.5 (n = 133466 ATAC-seq peaks) to the average accessibility profile of each of the three clusters. For clusters 1 and 2, the distribution of correlation coefficients was bimodal, corresponding to peaks that were highly correlated or anti-correlated with the profiles identified from HIRE2 to HIRE1 peaks. Plots extend from the data minima to the maxima; the white dot indicates the median, the box shows the interquartile range and whiskers extend to the most extreme data points.**d.** UpSet plot showing the top 1000 ATAC-seq peaks (ranked by their correlation coefficient) for each of the three clusters. There is no overlap between the top 1000 peaks for each cluster. **e.** Transcription factor motif enrichment heatmap. Shown are the log2-fold enrichments and -log10 (FDR) values for all transcription factor motifs with an FDR less than 0.001 in any of the three clusters (n = 382). Each row depicts one transcription factor motif. While there are many significantly enriched motifs for clusters 1 and 2, no specific motifs are enriched in cluster 3 or all residual ATAC-seq peaks. **f.** Histogram of the average motif hit rates per kilobase (kb). The 31 ATAC-seq peaks overlapping HIRE1 and HIRE2 were scanned for motif hits using all transcription factor motifs with an FDR less than 0.001 in any of the three clusters as input (see Methods). To select motifs with a high frequency of predicted binding sites, the average motif hit rates per kb were calculated and clustered into two sets with k-means clustering (k=2). All motifs from the set with higher hit-rates (n = 107) were used in a motif similarity analysis. **g.** Transcription factor motif enrichment heatmap. Shown are the log2-fold enrichments for all 107 transcription factor motifs with high hit-rates from **f** and an FDR less than 0.001 in any of the three clusters. The rows are ordered according to the motif similarity analysis shown in Supplementary Fig. 13. and yellow highlights indicate the different motif similarity clusters defined in Supplementary Fig. 13.

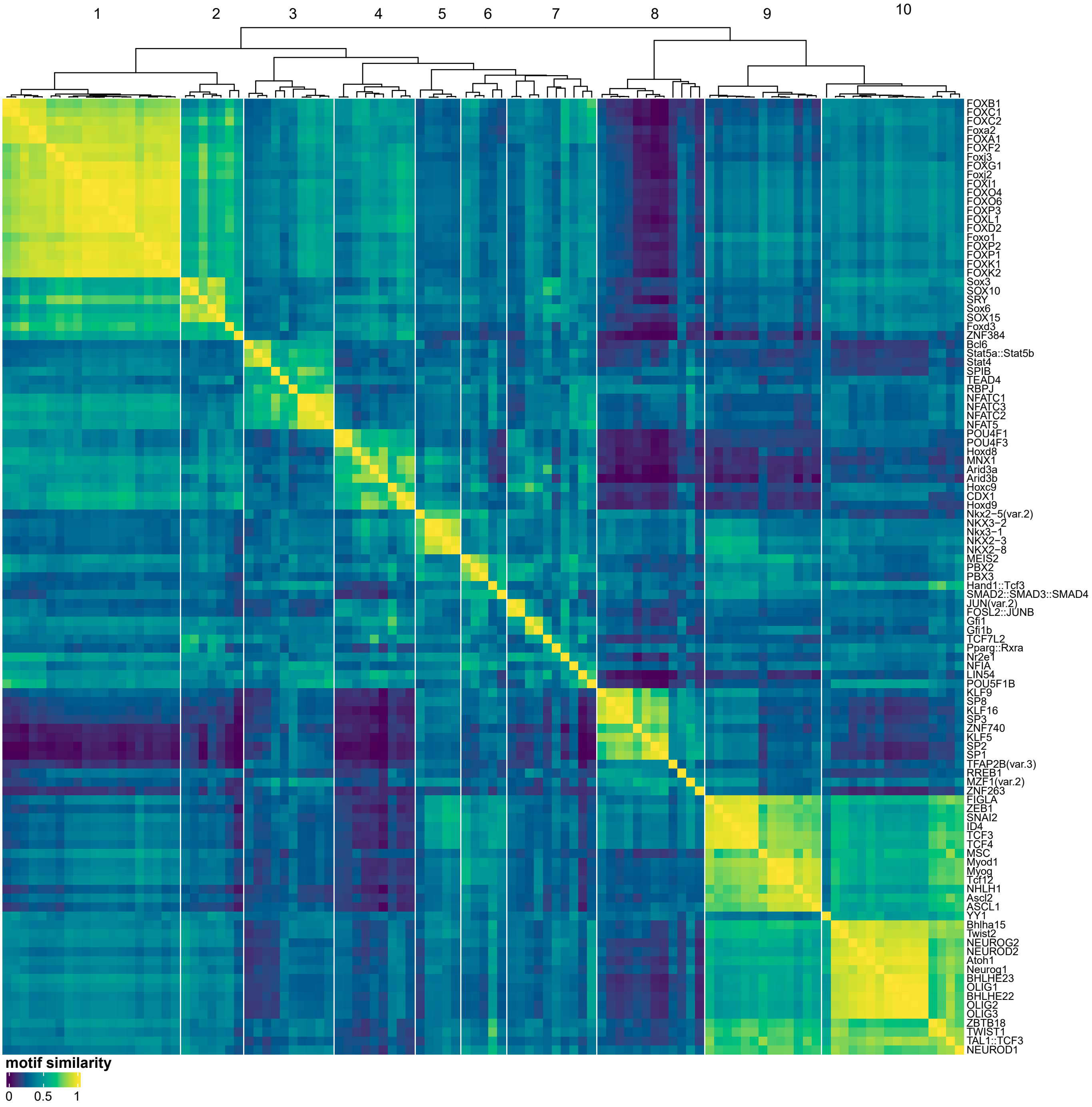
1
2
3
4
5
6
7
8
9
10
FOXB1
FOXC1
FOXC2
Foxa2
FOXA1
FOXF2
Foxj3
FOXG1
Foxj2
FOXI1
FOXO4
FOXO6
FOXP3
FOXL1
FOXD2
Foxo1
FOXP2
FOXP1
FOXK1
FOXK2
Sox3
SOX10
SRY
Sox6
SOX15
Foxd3
ZNF384
Bcl6
Stat5a::Stat5b
Stat4
SPIB
TEAD4
RBPJ
NFATC1
NFATC3
NFATC2
NFAT5
POU4F1
POU4F3
Hoxd8
MNX1
Arid3a
Arid3b
Hoxc9
CDX1
Hoxd9
Nkx2−5(var.2)
NKX3−2
Nkx3−1
NKX2−3
NKX2−8
MEIS2
PBX2
PBX3
Hand1::Tcf3
SMAD2::SMAD3::SMAD4
JUN(var.2)
FOSL2::JUNB
Gfi1
Gfi1b
TCF7L2
Pparg::Rxra
Nr2e1
NFIA
LIN54
POU5F1B
KLF9
SP8
KLF16
SP3
ZNF740
KLF5
SP2
SP1
TFAP2B(var.3)
RREB1
MZF1(var.2)
ZNF263
FIGLA
ZEB1
SNAI2
ID4
TCF3
TCF4
MSC
Myod1
Myog
Tcf12
NHLH1
Ascl2
ASCL1
YY1
Bhlha15
Twist2
NEUROG2
NEUROD2
Atoh1
Neurog1
BHLHE23
OLIG1
BHLHE22
OLIG2
OLIG3
ZBTB18
TWIST1
TAL1::TCF3
NEUROD1
motif similarity
0
0.5
1

Supplementary Fig. 13 Motif similarity analysis. All 107 transcription factor motifs with high hit-rates defined in Supplementary Fig. 12f were used to perform a motif similarity analysis using *monaLisa*[4]. See Methods for details.

Supplementary Table 1 Genomic locations of the 2,232 identified CNCC super-enhancers

The table contains the genomic locations (mm10) of all super-enhancers identified in CNCCs at E10.5 based on their H3K27ac signal.

Supplementary Table 2 SEs interacting with genes encoding for transcription factor genes differentially expressed in CNCCs subpopulations at E10.5.

The table contains the genomic coordinates (mm10) of the 147 super-enhancers (SEs) with at least one significant interaction to an expressed positional transcription factor (TF) gene as depicted in figure 1, including the information to which TF each SE is linked. In addition, the mean strengths of all interactions from the positional TF promoter to restriction fragment(s) that overlap with a SE are given for each population. The last column indicates the number of the k-means cluster as shown on the left side of the heatmap. The rows follow the same order as the heatmap of figure 1. Note that the table contains 148 unique SE-promoter pairs (rows) because one SE is shared by 2 different promoters (*Twist2* and *Hes6*), and thus appears twice.

Supplementary Table 3 Differential gene expression analysis. The table provides on five separate sheets the differential gene expression analysis between second pharyngeal arch (PA2) derived CNCCs at E10.5 and pinna derived CNCCs at E12.5, between PA2 derived CNCCs at E10.5 and pinna derived CNCCs at E14.5, between pinna derived CNCCs at E12.5 and pinna derived CNCCs at E14.5, between PA2 cells from *HIRE1*$^{del/del}$ and wild-type embryos at E10.5, and between PA3 cells from *HIRE1*$^{del/del}$ and wild-type embryos at E10.5. Each sheet contains the following information in the columns: Entrez gene ID, gene width, ENSEMBL gene ID, gene symbol, log2 fold change (column name logFC), mean expression level (log2 counts per million, column name logCPM), quasi-likehood F-test score (F), raw P value (PValue), and false discovery rate (FDR). P-values and FDR were calculated by *edgeR*[5]. See Methods for details on statistical analysis.

Supplementary Table 4 Transcription factor motif enrichment analysis. The table contains the results of the transcription factor motif analysis. Each row stands for one of the motifs of the JASPAR2018 database. The columns contain information about the motif ID, motif name, motif family, and motif class, as well as the log2-fold enrichments and -log10(FDR) values for each of the ATAC-seq peak clusters (as defined in Supplementary Fig. 12b-d) and all residual peaks. P-values and FDR were calculated by *monaLisa*[4]. See Methods for details.

Supplementary Table 5 Annotation of merged ATAC-seq peaks from PA2 derived CNCCs at E10.5 and pinna derived CNCCs at E12.5 overlapping the region spanning from HIRE2 to HIRE1 with predicted motifs. The number of occurrences of all transcription factor motifs with an FDR less than 0.001 in any of the three cluster profiles (Suppl. Fig. 8D) was predicted within each of the ATAC-seq peaks from second pharyngeal arch (PA2) derived cranial neural crest cells (CNCCs) at E10.5 and pinna derived CNCCs at E12.5, which overlap the region spanning from HIRE2 to HIRE1 (chr6:50789172-51087888, mouse GRCm38/mm10). See Methods for details.

Supplementary Table 6 List of selected representative motifs clustered by motif similarity analysis. Each row in the table stands for one of the motifs identified in the motif enrichment analysis (see Methods). The columns contain information about the motif similarity cluster that a motif was assigned to in Supplementary Fig. 13, the motif ID, motif name, motif family and motif class.

Supplementary Table 7 Sample information. The type of experiments performed on the different cell types, the protocols used for the libraries, the sample names, and for previously published datasets the GEO accession number are indicated.

Supplementary References

1. Donaldson IJ, *et al.* Genome-wide occupancy links Hoxa2 to Wnt-beta-catenin signaling in mouse embryonic development. *Nucleic Acids Res* **40**, 3990-4001 (2012).

2. Amin S, *et al.* Hoxa2 selectively enhances Meis binding to change a branchial arch ground state. *Dev Cell* **32**, 265-277 (2015).

3. Rijli FM, Mark M, Lakkaraju S, Dierich A, Dolle P, Chambon P. A homeotic transformation is generated in the rostral branchial region of the head by disruption of Hoxa-2, which acts as a selector gene. *Cell* **75**, 1333-1349 (1993).

4. Machlab D, Burger L, Soneson C, Rijli FM, Schubeler D, Stadler MB. monaLisa: an R/Bioconductor package for identifying regulatory motifs. *Bioinformatics*, (2022).

5. Robinson MD, McCarthy DJ, Smyth GK. edgeR: a Bioconductor package for differential expression analysis of digital gene expression data. *Bioinformatics* **26**, 139-140 (2010).
