# Supplementary material for: A multiple super-enhancer region establishes inter-TAD interactions and controls Hoxa function in cranial neural crest
Source: Nat Commun. 2023 Jun 5;14:3242. doi: 10.1038/s41467-023-38953-0 (PMC10241789; doi:10.1038/s41467-023-38953-0)
Supplement: Supplementary file 3 — Description of Additional Supplementary Files [file 41467_2023_38953_MOESM3_ESM.pdf]

Description of Additional Supplementary Files

Supplementary Data 1 Genomic locations of the 2,232 identified CNCC super-enhancers
The table contains the genomic locations (mm10) of all super-enhancers identified in CNCCs at E10.5 based on their H3K27ac signal.

Supplementary Data 2 SEs interacting with genes encoding for transcription factor genes differentially expressed in CNCCs subpopulations at E10.5.
The table contains the genomic coordinates (mm10) of the 147 super-enhancers (SEs) with at least one significant interaction to an expressed positional transcription factor (TF) gene as depicted in figure 1, including the information to which TF each SE is linked. In addition, the mean strengths of all interactions from the positional TF promoter to restriction fragment(s) that overlap with a SE are given for each population. The last column indicates the number of the k-means cluster as shown on the left side of the heatmap. The rows follow the same order as the heatmap of figure 1. Note that the table contains 148 unique SE-promoter pairs (rows) because one SE is shared by 2 different promoters (*Twist2* and *Hes6*), and thus appears twice.

Supplementary Data 3 Differential gene expression analysis. The table provides on five separate sheets the differential gene expression analysis between second pharyngeal arch (PA2) derived CNCCs at E10.5 and pinna derived CNCCs at E12.5, between PA2 derived CNCCs at E10.5 and pinna derived CNCCs at E14.5, between pinna derived CNCCs at E12.5 and pinna derived CNCCs at E14.5, between PA2 cells from $HIRE1^{del/del}$ and wild-type embryos at E10.5, and between PA3 cells from $HIRE1^{del/del}$ and wild-type embryos at E10.5. Each sheet contains the following information in the columns: Entrez gene ID, gene width, ENSEMBL gene ID, gene symbol, log2 fold change (column name logFC), mean expression level (log2 counts per million, column name logCPM), quasi-likehood F-test score (F), raw P value (PValue), and false discovery rate (FDR). P-values and FDR were calculated by *edgeR*[5]. See Methods for details on statistical analysis.

Supplementary Data 4 Transcription factor motif enrichment analysis. The table contains the results of the transcription factor motif analysis. Each row stands for one of the motifs of the JASPAR2018 database. The columns contain information about the motif ID, motif name, motif family, and motif class, as well as the log2-fold enrichments and -log10(FDR) values for each of the ATAC-seq peak clusters (as defined in Supplementary Fig. 12b-d) and all residual peaks. P-values and FDR were calculated by *monaLisa*[4]. See Methods for details.

Supplementary Data 5 Annotation of merged ATAC-seq peaks from PA2 derived CNCCs at E10.5 and pinna derived CNCCs at E12.5 overlapping the region spanning from HIRE2 to HIRE1 with predicted motifs. The number of occurrences of all transcription factor motifs with an FDR less than 0.001 in any of the three cluster profiles (Suppl. Fig. 8D) was predicted within each of the ATAC-seq peaks from second pharyngeal arch (PA2) derived cranial neural crest cells (CNCCs) at E10.5 and pinna derived CNCCs at E12.5, which overlap the region spanning from HIRE2 to HIRE1 (chr6:50789172-51087888, mouse GRCm38/mm10). See Methods for details.

Supplementary Data 6 List of selected representative motifs clustered by motif similarity analysis. Each row in the table stands for one of the motifs identified in the motif enrichment analysis (see Methods). The columns contain information about the motif similarity cluster that a motif was assigned to in Supplementary Fig. 13, the motif ID, motif name, motif family and motif class.

Supplementary Data 7 Sample information. The type of experiments performed on the different cell types, the protocols used for the libraries, the sample names, and for previously published datasets the GEO accession number are indicated.
