# Supplementary material for: A multiple super-enhancer region establishes inter-TAD interactions and controls Hoxa function in cranial neural crest
Source: Nat Commun. 2023 Jun 5;14:3242. doi: 10.1038/s41467-023-38953-0 (PMC10241789; doi:10.1038/s41467-023-38953-0)
Supplement: Supplementary file 11 — Reporting Summary [file 41467_2023_38953_MOESM11_ESM.pdf]

nature portfolio

Corresponding author(s): Filippo M. Rijli
Last updated by author(s): Apr 27, 2023

Reporting Summary

Nature Portfolio wishes to improve the reproducibility of the work that we publish. This form provides structure for consistency and transparency in reporting. For further information on Nature Portfolio policies, see our Editorial Policies and the Editorial Policy Checklist.

Statistics

For all statistical analyses, confirm that the following items are present in the figure legend, table legend, main text, or Methods section.

n/a	Confirmed	
☐	☒	The exact sample size (*n*) for each experimental group/condition, given as a discrete number and unit of measurement
☐	☒	A statement on whether measurements were taken from distinct samples or whether the same sample was measured repeatedly
☐	☒	The statistical test(s) used AND whether they are one- or two-sided<br>*Only common tests should be described solely by name; describe more complex techniques in the Methods section.*
☐	☒	A description of all covariates tested
☐	☒	A description of any assumptions or corrections, such as tests of normality and adjustment for multiple comparisons
☐	☒	A full description of the statistical parameters including central tendency (e.g. means) or other basic estimates (e.g. regression coefficient) AND variation (e.g. standard deviation) or associated estimates of uncertainty (e.g. confidence intervals)
☐	☒	For null hypothesis testing, the test statistic (e.g. *F*, *t*, *r*) with confidence intervals, effect sizes, degrees of freedom and *P* value noted<br>*Give P values as exact values whenever suitable.*
☒	☐	For Bayesian analysis, information on the choice of priors and Markov chain Monte Carlo settings
☒	☐	For hierarchical and complex designs, identification of the appropriate level for tests and full reporting of outcomes
☐	☒	Estimates of effect sizes (e.g. Cohen's *d*, Pearson's *r*), indicating how they were calculated

Our web collection on statistics for biologists contains articles on many of the points above.

Software and code

Policy information about availability of computer code

Data collection

Illumina NextSeq 500, Illumina HiSeq 2500, Illumina NovaSeq 6000, StepOnePlus Real-Time PCR System

Data analysis

ChIP-seq data analysis:
bowtie2 (version 2.4.2), Samtools (version 1.2), MACS2 (version 2.1.3.3), R (version 4.1.3), QuasR (version 1.34.0), Rbowtie (version 1.34.0), GenomicRanges (version 1.46.1), GenomeInfoDb (version 1.30.1), IRanges (version 2.28.0), S4Vectors (version 0.32.4), BiocGenerics (version 0.40.0), pyGenomeTracks (version 3.7)

ATAC-seq data analysis:
cutadapt (version 3.7), bowtie2 (version 2.4.2), Samtools (version 1.2), MACS2 (version 2.1.3.3), QuasR package (version 1.34.0), Rbowtie (version 1.34.0), GenomicRanges (version 1.46.1), GenomeInfoDb (version 1.30.1), IRanges (version 2.28.0), S4Vectors (version 0.32.4), BiocGenerics (version 0.40.0), pyGenomeTracks (version 3.7)

RNA-seq data analysis:
R (version 4.1.3), TxDb.Mmusculus.UCSC.mm10.knownGene package (version 3.4.7), BSgenome.Mmusculus.UCSC.mm10 (version 1.4.3), BSgenome (version 1.62.0), rtracklayer (version 1.54.0), Biostrings (version 2.62.0), XVector (version 0.34.0), Rhisat2 (version 1.10.0), QuasR (version 1.34.0), Rbowtie (version 1.34.0), GenomicRanges (version 1.46.1), GenomeInfoDb (version 1.30.1), IRanges (version 2.28.0), S4Vectors (version 0.32.4), BiocGenerics (version 0.40.0), GenomicFeatures (version 1.46.5), AnnotationDbi (version 1.56.2), cowplot (version 1.1.1), reshape2 (version 1.4.4), ggplot2 (version 3.3.5), RColorBrewer (version 1.1-3), circlize (version 0.4.14), ComplexHeatmap (version 2.10.0), SummarizedExperiment (version 1.24.0), Biobase (version 2.54.0), MatrixGenerics (version 1.6.0), matrixStats (version 0.61.0), edgeR (version 3.38.1), pyGenomeTracks (version 3.7)

Hi-C data analysis:
HiCUP (version 0.6.1), Juicer (version 1.6), HiCExplorer (version 3.7.2), pyGenomeTracks (version 3.7), hic2cool (version 0.8.3), FAN-C (version 0.9.26)

promoter-capture Hi-C data analysis:
CHiCAGO (version 1.24.0), ChiCMaxima (version 1.0): https://github.com/yousra291987/ChiCMaxima, HiCUP (version 0.6.1), pyGenomeTracks (version 3.7)

super-enhancer analysis:
MACS2 (version 2.1.3.3), R (version 4.1.3), BSgenome.Mmusculus.UCSC.mm10 (version 1.4.3), BSgenome (version 1.62.0), rtracklayer (version 1.54.0), Rsamtools (version 2.10.0), Biostrings (version 2.62.0), XVector (version 0.34.0), dplyr (version 1.0.8), GenomicInteractions (version 1.30.0), csaw (version 1.28.0), SummarizedExperiment (version 1.24.0), InteractionSet (version 1.22.0), TxDb.Mmusculus.UCSC.mm10.knownGene (version 3.10.0), MatrixGenerics (version 1.6.0), matrixStats (version 0.61.0), GenomicFeatures (version 1.46.5), AnnotationDbi (version 1.56.2), Biobase (version 2.54.0), QuasR (version 1.34.0), Rbowtie (version 1.34.0), GenomicRanges (version 1.46.1), GenomeInfoDb (version 1.30.1), IRanges (version 2.28.0), S4Vectors (version 0.32.4), BiocGenerics (version 0.40.0), circlize (version 0.4.14), ComplexHeatmap (version 2.10.0), data.table (version 1.14.2), GenomicInteractions (version 1.30.0)

CTCF motif occurrences:
FIMO (version 5.4.1), JASPAR2022 (version 0.99.7)

transcription factor motif enrichment analysis:
MACS2 (version 2.1.3.3), R (version 4.0.5), BSgenome.Mmusculus.UCSC.mm10 (version 1.4.0), BSgenome (version 1.58.0),TFBSTools (version 1.28.0), TxDb.Mmusculus.UCSC.mm10.knownGene package (version 3.4.7), GenomicFeatures (version 1.42.3), AnnotationDbi (version 1.52.0), Biobase (version 2.50.0), rtracklayer (version 1.50.0 , limma (version 3.46.0), Rsamtools (version 2.6.0), Biostrings (version 2.58.0), XVector (version 0.30.0), QuasR (version 1.34.0), Rbowtie (version 1.30.0), GenomicRanges (version 1.42.0), GenomeInfoDb (version 1.26.7), IRanges (version 2.24.1), S4Vectors (version 0.28.1), BiocGenerics (version 0.36.1), SingleCellExperiment (version 1.12.0), SummarizedExperiment (version 1.20.0), MatrixGenerics (version 1.2.1), matrixStats (version 0.58.0), JASPAR2018 (version 1.1.1), monaLisa (version 0.1.40), UpSetR (version 1.4.0), vioplot (version 0.3.6), zoo (version 1.8-9), sm (version 2.2-5.6), ComplexHeatmap (version 2.6.2)

Selection of representative motifs:
R (version 4.2.2), circlize (version 0.4.15), JASPAR2018 (version 1.1.1), BSgenome.Mmusculus.UCSC.mm10 (version 1.4.3), rtracklayer (version 1.58.0), BioStrings (version 2.66.0), XVector (version 0.38.0), TFBSTools (version 1.36.0), SummarizedExperiment (version 1.28.0), Biobase (version 2.58.0), GenomicRanges (version 1.50.1), GenomInfoDb (version 1.34.3), IRanges (version 2.32.0), S4Vectors (version 0.36.0), BiocGenerics (version 0.44.0), MatrixGenerics (version 1.10.0), matrixStats (version 0.63.0), ComplexHeatmap (version 2.14.0)

Reference genome GRCm38/mm10

MA Cell Sorter Software (version 3.2.0), BD FACS Software (version 1.2.0.142)

For manuscripts utilizing custom algorithms or software that are central to the research but not yet described in published literature, software must be made available to editors and reviewers. We strongly encourage code deposition in a community repository (e.g. GitHub). See the Nature Portfolio guidelines for submitting code & software for further information.

Data

Policy information about availability of data

All manuscripts must include a data availability statement. This statement should provide the following information, where applicable:

- Accession codes, unique identifiers, or web links for publicly available datasets
- A description of any restrictions on data availability
- For clinical datasets or third party data, please ensure that the statement adheres to our policy

All raw sequencing data and processed data generated in this study have been deposited and are publicly available from the Gene Expression Omnibus (GEO) under GEO Series accession number GSE211904 (all data SuperSeries), GSE211899 (ATAC-seq), GSE211900 (ChIP-seq), GSE211901 (Hi-C), GSE211902 (PCHi-C) and GSE211903 (RNA-seq).

In addition, we used following public sequencing datasets published in Minoux et al. 2017 and available through GEO from mouse E10.5 cranial neural crest cell subpopulations (GSE89437).

ChIP-seq peaks for Hoxa2 in PA2 at E11.5 were obtained from Donaldson et al. 2012 (https://doi.org/10.1093/nar/gkr1240), and ChIP-seq peak data for Pbx and Meis in PA2 at E11.5 were obtained from Amin et al., 2015 (https://doi.org/10.1016/j.devcel.2014.12.024).

Furthermore, we used following public databases:
UCSC (mm10 reference genome assembly, gene annotation), JASPAR 2018 (vertebrate transcription factor motifs), JASPAR 2022 (vertebrate transcription factor motifs), ENCODE Blacklist (genomic regions that have anomalous, unstructured, or high signal in next-generation sequencing experiments independent of cell line or experiment)

Human research participants

Policy information about studies involving human research participants and Sex and Gender in Research.

Reporting on sex and gender	N/A
Population characteristics	N/A
Recruitment	N/A
Ethics oversight	N/A

Note that full information on the approval of the study protocol must also be provided in the manuscript.

Field-specific reporting

Please select the one below that is the best fit for your research. If you are not sure, read the appropriate sections before making your selection.

☒ Life sciences ☐ Behavioural & social sciences ☐ Ecological, evolutionary & environmental sciences

For a reference copy of the document with all sections, see nature.com/documents/nr-reporting-summary-flat.pdf

Life sciences study design

All studies must disclose on these points even when the disclosure is negative.

Sample size	We chose samples sizes regarding replicates based on the standards of the field (e.g. the ENCODE Data standards, ENCSR534HMF, ENCSR068YGC). We did not perform statistical tests to predetermine samples sizes.
Data exclusions	No data was excluded.
Replication	As for qPCR and histological experiments, all the experiments are based on at least three biologically independent replicates (embryos). Each attempt of replication was successful and reproducibility among replicates was very high.<br><br>As for Sequencing experiments all have at least 2-3 biologically independent replicates, with one exception (see below, and see deposited data and methods).<br>All RNA-seq experiments were performed in biologically independent triplicates.<br>The ATAC-seq experiments performed for this study at E10.5 have 2 biologically independent replicates, at E12.5 have 3 biologically independent replicates and at E14.5 have 5 biologically independent replicates.<br>All ChIP-seq experiments on histone marks have 2 biologically independent replicates. CTCF ChIP experiments on cells of the mandibular prominence (Md) and second pharyngeal arch (PA2) at E10.5 have one replicate each. The pattern of CTCF binding in these two samples was essentially identical.<br>Hi-C experiments at E12.5 and E14.5 were performed in biologically independent duplicates. Hi-C experiments of mESCs were performed in duplicate and merged for data analysis. HI-C experiments on cells of the mandibular prominence (Md) and second pharyngeal arch (PA2) at E10.5 have one replicate each.<br>All promoter-capture Hi-C experiments have 2 biologically independent replicates.<br>Overall, reproducibility among replicates for all experiments was very high.
Randomization	For Mouse experiments, each biological replicate was prepared from independent litter mates. All mice and embryos used were raised and/or prepared under the same conditions and randomly allocated to the different experimental groups.
Blinding	No blinding was performed.

Reporting for specific materials, systems and methods

We require information from authors about some types of materials, experimental systems and methods used in many studies. Here, indicate whether each material, system or method listed is relevant to your study. If you are not sure if a list item applies to your research, read the appropriate section before selecting a response.

Materials & experimental systems

n/a	Involved in the study
[ ]	[x] Antibodies
[ ]	[x] Eukaryotic cell lines
[x]	[ ] Palaeontology and archaeology
[ ]	[x] Animals and other organisms
[x]	[ ] Clinical data
[x]	[ ] Dual use research of concern

Methods

n/a	Involved in the study
[ ]	[x] ChIP-seq
[ ]	[x] Flow cytometry
[x]	[ ] MRI-based neuroimaging

Antibodies

Antibodies used	Rabbit polyclonal anti-H3K4me2 antibody, Millipore 07-030<br>Rabbit polyclonal anti-H3K27me3 antibody, Millipore 07-449<br>Rabbit polyclonal anti-H3K27ac antibody, Abcam ab4729<br>Rabbit polyclonal anti-CTCF antibody, Cell Signaling CST 2899<br>Sheep polyclonal anti-Digoxigenin-AP, Fab-Fragmente, Roche 1109327491
Validation	anti-H3K4me2 antibody, Millipore 07-030: validated by the vendor by Western blotting (WB), ChIP-seq, immunocytochemistry (ICC)<br>anti-H3K27me3 antibody, Millipore 07-449: validated by the vendor by WB, immunohistochemistry (IHC), immunoprecipitaiton (IP)<br>anti-H3K27ac antibody, Abcam ab4729: validated by the vendor by WB, ChIP, ICC, IHC<br>anti-CTCF antibody, Cell Signaling CST 2899: validated by the vendor by WB, ChIP, IP, Immunofluorescence (IF)<br>anti-Digoxigenin-AP, Fab-Fragmente, Roche 11093274910: validated by the vendor. Dilution 1:2000.

Eukaryotic cell lines

Policy information about cell lines and Sex and Gender in Research

Cell line source(s)	The E14 mouse embryonic stem cell line (mESCs) for HiC-seq was provided by Luca Giorgetti (FMI, Basel, Switzerland).
Authentication	Cell line has not been authenticated.
Mycoplasma contamination	Cell line tested negative for mycoplasma.
Commonly misidentified lines<br>(See ICLAC register)	No commonly misidentified lines were used.

Animals and other research organisms

Policy information about studies involving animals; ARRIVE guidelines recommended for reporting animal research, and Sex and Gender in Research

Laboratory animals	All mouse lines used, including the newly generated HIRE1 and HIRE2 lines, were maintained on a mixed background (C57Bl/6J; CD1). Mice were housed in a 12 h light:dark cycle and given ad libitum access to food and water for the duration of the study. Ambient temperature is 22 +/-2 °C and humidity is maintained at 45-65%. For breeding, one or two female mice were introduced into a cage with a single male and monitored for timed pregnancies. Noon of the day of the vaginal plug was considered as E0.5. All mice used for breedings were at least 8 weeks old and not older than 6 month.<br><br>Mouse embryos were collected at stage E8.5, E9.5, E10.5, E12.5 or E14.5 depending on the downstream experiment (e.g. in situ hybridization, capture Hi-C, etc.).
Wild animals	No involvement of wild animals.
Reporting on sex	Sex was not considered in the study design and findings apply to both sexes. Embryos with different sexes were pooled to carry out molecular analysis including sequencing. Our study aims to uncover general molecular regulatory mechanisms during facial morphogenesis for which sex is considered irrelevant.
Field-collected samples	No involvement of field-collected samples
Ethics oversight	All animal experiments were approved by the Basel Cantonal Veterinary Authorities under permit 2670 and conducted in accordance with the Guide for Care and Use of Laboratory Animals.

Note that full information on the approval of the study protocol must also be provided in the manuscript.

ChIP-seq

Data deposition

☒ Confirm that both raw and final processed data have been deposited in a public database such as GEO.

☒ Confirm that you have deposited or provided access to graph files (e.g. BED files) for the called peaks.

Data access links<br>*May remain private before publication.*	GEO accession number GSE211904 (https://www.ncbi.nlm.nih.gov/geo/query/acc.cgi?acc=GSE211904)<br>Reviewer access token: mduzgeaelfodjcf . Data are publicly available as of today April 27, 2023.
Files in database submission	fastq and bigwig files for the following samples:<br>H3K27ac_wt_E12.5_Pinna<br>H3K27ac_rep2_wt_E12.5_Pinna<br>H3K4me2_wt_E12.5_Pinna<br>H3K4me2_rep2_wt_E12.5_Pinna<br>H3K27me3_wt_E12.5_Pinna<br>H3K27me3_rep2_wt_E12.5_Pinna<br>H3K27ac_wt_E14.5_Pinna<br>H3K27ac_rep2_wt_E14.5_Pinna<br>H3K4me2_wt_E14.5_Pinna<br>H3K4me2_rep2_wt_E14.5_Pinna<br>H3K27me3_wt_E14.5_Pinna<br>H3K27me3_rep2_wt_E14.5_Pinna<br>CTCF_E10_PA2<br>CTCF_E10_Md
Genome browser session<br>(e.g. UCSC)	bigwig files used for figures are deposited in GEO. Genome browser views were generated with pyGenomeTracks (version 3.7)

Methodology

Replicates	All ChIP-seq samples generated for this study have two replicates, except CTCF ChIP-seq (see above, replication section).
Sequencing depth	ChIP-seq libraries were sequenced on an Illumina HiSeq 2500 machine (50bp read length, single end). The total sequencing depth for each sample was between 30M to 60M reads, and more than 90% reads had high quality mapping to mm10.
Antibodies	Rabbit polyclonal anti-H3K4me2 antibody, Millipore 07-030<br>Rabbit polyclonal anti-H3K27me3 antibody, Millipore 07-449<br>Rabbit polyclonal anti-H3K27ac antibody, Abcam ab4729<br>Rabbit polyclonal anti-CTCF antibody, Cell Signaling CST 2899
Peak calling parameters	Reads were mapped to the reference genome using bowtie2 with default settings (version 2.4.2) and converted to bam files using samtools (version 1.2).<br><br>Peak calling for super-enhancer analysis:<br>Raw reads from all E10.5 samples (GSM2371717, GSM2371718, GSM2371719, GSM2371720) were mapped to the reference genome using bowtie2 with default settings (version 2.4.2) (Langmead, 2012;Langmead, 2019) and converted to bam files using samtools (version 1.2) (Danecek, 2021, Li, 2009). Bam files of the H3K27ac ChIP-seq alignments were merged using RSamtools (version 2.10.0). Then, peaks were called using MACS2 (version 2.1.3.3) (Zhang 2008) with the options --nomodel, --shift 0, --extsize 141, --keep-dup all, --qvalue 0.001, --cutoff-analysis. The extension size had been determined previously based on cross correlation using the csaw Bioconductor package (version 1.28.0) (Lun, 2016). Peaks that overlapped with promoter regions, i.e. 2500bp upstream or downstream of the transcription start sites of the UCSC known genes (TxDb.Mmusculus.UCSC.mm10.knownGene, v. 3.10.0), or peaks overlapping with blacklisted regions (Amemiya, 2019) were removed. Only peaks located on chromosomes 1-19 or the X-chromosome were kept.
Data quality	All samples were quality controlled by qQCReport function of QuasR package version 1.34.0. GC bias was assessed.
Software	bowtie2 (version 2.4.2), Samtools (version 1.2), MACS2 (version 2.1.3.3), R (version 4.1.3), QuasR (version 1.34.0), Rbowtie (version 1.34.0), GenomicRanges (version 1.46.1), GenomeInfoDb (version 1.30.1), IRanges (version 2.28.0), S4Vectors (version 0.32.4), BiocGenerics (version 0.40.0), pyGenomeTracks (version 3.7)

Flow Cytometry

Plots

Confirm that:

- [x] The axis labels state the marker and fluorochrome used (e.g. CD4-FITC).
- [x] The axis scales are clearly visible. Include numbers along axes only for bottom left plot of group (a 'group' is an analysis of identical markers).
- [x] All plots are contour plots with outliers or pseudocolor plots.
- [x] A numerical value for number of cells or percentage (with statistics) is provided.

Methodology

Sample preparation	Dissected tissue from E10.5 and E12.5 embryos was kept in 1× PBS on ice, then treated with 0.5% trypsin/1× EDTA at 37 °C for 10 minutes and immediately put on ice. Dissected tissue from E14.5 embryos was kept in 1× PBS on ice, then treated with papain digestion mix (10 mg ml−1 papain, 2.5 mM cysteine, 10 mM HEPES (pH 7.4), 0.5 mM EDTA and 0.9× DMEM) for 7 min at 37 °C and immediately put on ice. The tissue was rinsed once in ice-cold 1× DMEM/10% FBS, followed by two washes in ice-cold 1× DMEM. Tissue was dissociated by pipetting. Cranial NCCs from embryos with genotype Wnt1::Cre;ROSARFP or Hoxa2EGFP/EGFP were either fixed with formaldehyde before being filtered and collected by FACS, i.e. for ChIP-seq, Hi-C and promoter-capture Hi-C, or filtered and collected by FACS directly, i.e. for ATAC- and RNA-seq. Further processing of cells was adapted depending on downstream application (e.g. RNA-seq, ATAC-seq, ChIP-seq, Hi-C, promoter-capture Hi-C).
Instrument	BD Influx and Sony MA900
Software	BD FACS Software and MA Cell Sorter Software
Cell population abundance	Signal positive fractions were always determined by comparing against equivalent tissues from negative control embryos which don't express fluorescent markers. Negative control embryos were prepared from the same litter as signal positive embryos.
Gating strategy	Cells were preliminary selected by scatter areas (FSC-A vs SSC-A). Single cells were discriminated from doublets by firstly assessing forward scatter (FSC-W vs FSC-H) and next by assessing side scatter (SSC-W vs SSC-H). Subsequently cells were sorted based on fluorescent markers of interest.

- [x] Tick this box to confirm that a figure exemplifying the gating strategy is provided in the Supplementary Information.
